# B Vitamins and Incidence of Advanced Age-Related Macular Degeneration: The Alienor Study

**DOI:** 10.3390/nu14142821

**Published:** 2022-07-08

**Authors:** Bénédicte M. J. Merle, Stéphanie Barthes, Catherine Féart, Audrey Cougnard-Grégoire, Jean-François Korobelnik, Marie-Bénédicte Rougier, Marie-Noëlle Delyfer, Cécile Delcourt

**Affiliations:** 1 Institut National pour la Santé et la Recherche Médicale (INSERM), Bordeaux Population Health (BPH), UMR1219, University of Bordeaux, 33000 Bordeaux, France; bastephanie@hotmail.fr (S.B.); catherine.feart-couret@u-bordeaux.fr (C.F.); audrey.cougnard-gregoire@u-bordeaux.fr (A.C.-G.); jean-francois.korobelnik@chu-bordeaux.fr (J.-F.K.); marie-benedicte.rougier@chu-bordeaux.fr (M.-B.R.); marie-noelle.delyfer@chu-bordeaux.fr (M.-N.D.); cecile.delcourt@u-bordeaux.fr (C.D.); 2 Department of Ophtalmologie, Centre Hospitalier Universitaire de Bordeaux, 33000 Bordeaux, France

**Keywords:** age-related macular degeneration, vitamins B, folate, epidemiology, nutrition, cohort, risk, population

## Abstract

B vitamins may protect against age-related macular degeneration (AMD). We evaluated the associations of dietary intake and serum vitamins with the incidence of advanced AMD in the Alienor study. The Alienor study is a prospective population-based cohort of 963 residents of Bordeaux, France, who were 73 years or older at baseline (2006–2008). Examinations were performed every two years over an eight-year period. The incidence of AMD is based on retinal fundus photographs and spectral-domain optical coherence tomography examinations. Among the 861 included participants, 93 developed incident AMD during a median follow-up time of 9.8 years. Participants with normal serum folate (≥10 nmol/L) significantly had a 51% reduced risk for AMD in the fully adjusted Cox model (HR, 0.49 [95% CI, 0.25–0.95], *p* = 0.036). Participants with a higher dietary intake of B5 and B6 vitamins had a lower risk for developing AMD of up to 28% (HR, 0.72 for 1-SD increase [0.53–0.99], *p* = 0.049; HR, 0.90 [0.81–0.99], *p* = 0.049, respectively). This cohort study of older adults suggests a strong association between a normal serum folate status, a high dietary intake of B5 and B6 and a lower risk for developing advanced AMD. Adopting a healthy diet rich in B vitamins may help to reduce vision loss due to AMD.

## 1. Introduction

Age-related macular degeneration (AMD) is the leading cause of central vision loss in industrialized countries [1]. It arises from a complex interplay among aging, genetic susceptibility, and lifestyle factors [1,2]. Advanced forms of the disease, neovascular or atrophic AMD, associated with visual impairment, are generally preceded by early stages. While no treatment is currently available for atrophic AMD, effective but costly treatments are available for the neovascular form [1]. Attention to modifiable risk factors is of utmost importance to reduce progression to advanced AMD and associated medical and societal burdens.

Nutrition is a well-known lifestyle factor linked to AMD. Epidemiological studies have reported a reduced risk of AMD associated with high consumption of antioxidants including lutein [3,4,5,6,7], omega-3 polyunsaturated fatty acids [7,8] and, recently, with a high adherence to a Mediterranean diet [9,10,11]. Other nutritional factors have not been extensively explored, suggesting that the additional analysis of biologically active nutrients may provide more insights into the molecular basis of the disease. In particular, epidemiologic data related to dietary intake and circulating status of B vitamins are scarce.

Low B vitamin status can increase the risk of developing degenerative diseases such as cardiovascular diseases, cognitive diseases or osteoporosis [12]. B vitamins (B6, folate, and B12) regulate homocysteine levels and hyperhomocysteinemia is a potential risk factor for AMD [13,14,15,16]. Through the one carbon metabolism, B vitamins also play a central role in the methylation and synthesis of DNA and its repair and replication [17,18].

Epidemiological studies have reported associations between higher intakes of vitamin B6 [19] and folate [7,20,21] and a lower risk for advanced AMD. To note, these studies have been conducted in countries with systematic foods fortification with folic acid and are mainly cross-sectional or case–control studies. One randomized controlled trial showed that a daily supplementation of folic acid and vitamins B6 and B12 reduced the risk of AMD by 40% [22]. Associations of lower concentrations of plasma folate [23] and vitamin B12 [23,24] with increased risk of AMD have also been reported. Further studies measuring both diet and circulating B vitamins exposures and using a prospective design are required to strengthen the potential beneficial effect of B vitamins in AMD development.

We therefore evaluated the associations between dietary and serum B vitamins and the incidence of advanced AMD in a population-based prospective study of French older adults.

## 2. Materials and Methods

### 2.1. Study Population

The Alienor Study is an ongoing prospective population-based study aiming at assessing the associations of age-related eye diseases with nutritional factors and other major determinants of eye disease [25].

Participants were recruited from a population-based study on the vascular risk factors for dementia, the Three-City (3C) Study [26]. The Alienor Study consists of eye examinations, which were offered to all participants of the 3C cohort in Bordeaux since the 3C third follow-up (http://www.alienor-study.com/langue-english-1.html, accessed on 1 June 2022). Detailed characteristics of the participants have been described elsewhere [25].

At Alienor baseline (2006–2008), 963 participants, aged 73 years or more, were interviewed and underwent an ophthalmological examination [25]. Of these, 395, 624, 614, 513, and 435 were reexamined at the Alienor ancillary study’s (2008–2009), first (2009–2010), second (2011–2012), third (2013–2015) and fourth (2015–2017) follow-up visits, respectively (Figure S1). The design was approved by the Ethical Committee of Bordeaux (Comité de Protection des Personnes Sud-Ouest et Outre-Mer III) in May 2006. All participants provided written informed consent in accordance with the Declaration of Helsinki to participate in the study.

### 2.2. Eye Examination

The eye examinations took place in the Department of Ophthalmology of the University Bordeaux Hospital. They included a recording of ophthalmic history, measures of visual acuity and two 45° nonmydriatic color retinal photographs (TRC NW6S; Topcon, Tokyo, Japan) [25]. In addition, from the first follow-up visit, a Spectral-Domain Optical Coherence Tomography (SD-OCT) examination of the macula and the optic nerve was performed using Spectralis (Software Version 5.4.7.0; Heidelberg Engineering, Heidelberg, Germany). The same experienced technician performed all SD-OCT assessments. In addition, from the second visit, for participants who were not able to come to the hospital, the eye examination took place at home and 40° retinal photographs were taken using a digital nonmydriatic portable retinograph.

### 2.3. AMD Classification

Retinal photographs of both eyes were graded by two trained graders and were interpreted according to the International Classification and to a modification of the grading scheme used in the Multi-Ethnic Study of Atherosclerosis for drusen size, location and area [27,28]. SD-OCT macular scans were interpreted for signs of retinal atrophy and neovascular AMD. Finally, classification of AMD was performed by retina specialists, using all available information (ophthalmological history and treatments, retinal photographs, SD-OCT scans).

Neovascular AMD included serous or hemorrhagic detachment of the retinal pigment epithelium (RPE) or sensory retina, subretinal or sub-RPE hemorrhages, and fibrous scar tissue. Geographic atrophy was defined as a discrete area of retinal depigmentation, 175 µm in diameter or larger, characterized by a sharp border and the presence of visible choroidal vessels. Early AMD was classified in two groups (in the absence of advanced AMD): early AMD 1 (soft distinct drusen without pigmentary abnormalities or pigmentary abnormalities without large drusen (>125 μm)) and early AMD 2 (soft indistinct drusen and/or reticular pseudodrusen and/or soft distinct drusen associated with pigmentary abnormalities (hyper- or hypopigmentation)). Soft distinct and indistinct drusen were larger than 125 μm in diameter with a uniform density and sharp edges, or decreasing density from the centre outwards and fuzzy edges, respectively. Pigmentary abnormalities were defined as areas of hyperpigmentation and/or hypopigmentation (without visibility of choroidal vessels). No AMD was defined by the absence of early AMD and advanced AMD.

In addition, SD-OCT macular scans (vertical and horizontal lines, macular volume) were interpreted for signs of retinal atrophy and neovascular AMD (subretinal fluid, subretinal tissue, pigment epithelium detachment, intra-retinal fluid). Finally, classification of atrophic and neovascular AMD was based on all available information (ophthalmological history and treatments, retinal photographs, SD-OCT scans).

At each visit, each eye was classified according to one of the following exclusive groups: no AMD, early AMD1, early AMD2 and advanced AMD. None of the people involved in the classification of AMD had any access to B vitamins measurements at any time of the study. AMD was classified masked to B vitamin status.

### 2.4. Event of Interest and Time Axis

Incidence of advanced AMD was defined as the participants progressing from no or early AMD at Alienor baseline to advanced AMD in either eye at any time-point during the study period, or as the participants with advanced AMD diagnosed at Alienor baseline declaring a date of AMD diagnosis between exposure measurement (diet questionnaires 2001) and Alienor baseline (Figure S1).

The date of occurrence of advanced AMD was calculated as the midpoint of the interval between the last visit without advanced AMD and the first visit with advanced AMD. Follow-up ended at the date of occurrence of advanced AMD, or the date of the last gradable examination. Individuals with advanced AMD or no gradable eyes at baseline were excluded from the analysis.

In the present analysis, time origin corresponds to dietary measurements (3C 1st follow-up) and endpoint time corresponds to the last patient’s visit. Time axis is time since dietary measurements.

### 2.5. Dietary Assessment

Participants were visited at home by a specifically trained dietician who administered a 148-item validated food frequency questionnaire (FFQ) and a 24 h dietary recall [29]. The dietician registered all the meals and beverages consumed in the 24 h period before the individual awoke on the day of the interview. None were recorded of weekend days. Quantities were assessed according to a book of photographs containing 236 foods or beverages [30]. The same dietician then entered the data of the 24 h recall software (Bilnut; Nutrisoft, Cerelles, France) to obtain an estimate of the daily nutrient intake of each participant. Food composition tables for France are included in this software [31,32,33]. The 24 h recall was used to estimate intake of vitamins B1, B2, B3, B5, B6, folate, B12 and total energy intake (TEI, kcal/day) and alcohol consumption (g/day) was assessed using the FFQ.

Dietary intake of B vitamins was estimated from food only. At the time of data collection, the contribution of supplemental folic acid (fortified foods or supplements) to total folate intake was very low and virtually all folic acid is of natural origin in French populations [34].

### 2.6. Serum Measurements

Serum measurements were determined from fasting blood samples collected at the 3C baseline visit into heparinized evacuated tubes and centrifuged at 1000× *g* for 15 min and stored at −80 °C until determinations.

Serum pyridoxine (vitamin B6) concentrations (nmol/L) were measured with liquid chromatography coupled to tandem mass spectrometry at CERBA laboratory (Saint Ouen l’Aumône, France). Serum folate (vitamin B9, nmol/L) and serum cobalamin (vitamin B12, pmol/L) concentrations were measured with Chemiluminescence immunoassay (Abbott Architect i2000SR) at EXALAB laboratory (Le Haillan, France). None of the people involved in the determination had any access to ocular clinical findings or genetic data at any time of the study.

### 2.7. Other Variables

Age, sex, smoking and physical activity were measured using self-reported questionnaires and body mass index (BMI: weight (kg)/height^2^ (m^2^)) was measured at 3C study baseline [25].

AMD nutritional supplement use was evaluated from the 3C study’s first follow-up to the last Alienor follow-up visit. Participants who reported taking vitamins, minerals, or AMD supplements at least once during this period were considered as users of AMD nutritional supplements.

High-density lipoprotein (HDL) concentrations were measured at the Biochemistry Laboratory of the University Hospital of Dijon (Dijon, France) using routine enzymatic techniques.

Genotyping was performed on DNA extracted from leukocytes at the 3C study baseline and kept frozen at −80 °C. Centralized facilities for genotyping were provided by Lille Genopôle, and a genome-wide scan was performed at Lille Genopôle [35]. The genetic risk score is based on Fritsche et al.’s paper [36]. The score corresponds to the sum of corresponding beta multiplied by the number of minor alleles for each single nucleotide polymorphism (SNP). The betas used are calculated from the fully conditioned odds ratios in the Fritsche’s paper. Due to the high number of missing data for the three following SNPs: *TRPM3* rs71507014, *CNN2* rs67538026 and *MMP9* rs142450006, these SNPs have been excluded from the risk score calculation.

The present genetic risk score is based on 49 SNPs and was calculated for all participants who had available data for at least five of the major AMD-related genes (*CFH* rs10922109, *CFH* rs570618, *C2* rs11603772, *C3* rs2230199 and *ARMS2* rs3750846).

### 2.8. Statistical Analysis

Each B vitamin was described by its mean and standard deviation (SD) and distribution was represented using a violin plot. Recommended Dietary Allowance (RDA) and 2/3 of RDA for the French population 65 years and over was displayed [34]. The recommended dietary allowance (RDA) corresponds to average daily level of intake sufficient to meet the nutrient requirements of nearly all (97–98%) healthy people. RDA is based on scientific knowledge. The RDA is estimated from the estimated average requirement (EAR) plus two standard deviations (SD) to determine the intake that covers the requirement of 97 to 98% of the population. The SDs most often estimated 15% of the EAR. Thus, we also represented the 2/3 RDA level in our graph as a proxy for the EAR.

The adjusted associations of B vitamins with incidence of advanced AMD were independently estimated using Cox proportional hazards models.

Potential confounders were identified from the literature and associations reported in the Alienor study: age, sex, smoking status, HDL-cholesterol, genetic risk score, oral supplementation for AMD, BMI and physical activity. In addition, all analyses of dietary B vitamins were adjusted for TEI, while analyses of serum B6 and B12 vitamins were adjusted for alcohol consumption.

In all Cox models, the proportional hazard assumptions were checked and satisfied using Schoenfeld’s residuals. The log linearity for all quantitative variables was tested using penalized splines with four degrees of freedom. Linearity was confirmed for all B vitamin variables, except for serum folate. To account for the nonlinear effects of folate variables, we used penalized splines with four degrees of freedom (p-spline function in the coxph function of R) [37,38]. Then, we used folate as a categorical variable using clinical cutoffs: normal (≥10 nmol/L) versus deficient (<10 nmol/L) [39]. Serum vitamin B6 and B12 were also used as categorical variables using clinical cutoffs of 20 nmol/L [40] and 185 pmol/L [41], respectively.

We used two-sided *p* values with a α = 0.05 threshold for statistical significance and R software (v3.6.1; R Core Team, Boston, MA, USA) for all analyses.

## 3. Results

### 3.1. Characteristics of the Sample

Among the 963 participants included in the Alienor study, 102 were excluded from the statistical analysis: 22 had advanced AMD at baseline, one had missing AMD status, 45 had missing data for diet exposure and 34 had no follow-up data (Figure S2). Characteristics of included and excluded participants were not different except for age, physical activity and genetic risk score. Included participants tended to be younger and to have a lower genetic risk score than excluded participants (Table S1).

Among the 861 included participants, mean age at baseline was 74.7 years, 530 (61.6%) were women and 93 (10.8%) developed advanced AMD during a median follow-up time of 9.8 years (range, 4.6 to 14.6 years).

Participants who developed advanced AMD more often tended to be women, more advanced in age, more frequent users of AMD supplements, with higher HDL-cholesterol levels and a higher genetic risk score compared to other participants (Table 1).

### 3.2. Description of Vitamin B Dietary Intake

Figure S3 displays dietary intakes at inclusion for each vitamin B according to gender. For dietary intake of B1 and B3 vitamins, approximatively half of the sample reached the RDA and less than 25% reported an intake below 2/3 of the RDA, regardless of gender. For vitamin B2, about 25% of the sample reached the RDA; 25% of men and 40% of women reported an intake below 2/3 of the RDA. For dietary intake of vitamin B5, less than 20% of the sample reached the RDA and around 35–40% reported an intake below 2/3 of the RDA, regardless of gender. For vitamin B6, about 40% of men and 25% of women reached the RDA. Approximately 25% of the sample was below 2/3 of the RDA. For vitamin B9 and B12, more than 40 to 50% of men reported intake above the RDA and around 25% below 2/3 of the RDA. Among women, around 25% reached the RDA and approximately 50% was below 2/3 of the RDA.

### 3.3. Multivariate Associations between Dietary Intake of B Vitamins and Risk of AMD

The relationship between dietary B vitamins and the risk of AMD did not depart significantly from log-linearity (Figure S4). At any time after baseline, the hazard of advanced AMD onset was significantly 28% lower when the intake of vitamin B5 was 1-SD (1.84 mg/day) higher, after multivariate adjustment. Similarly, the hazard of advanced AMD onset was significantly 10% lower when intake of vitamin B6 was 1-SD (0.60 mg/day) higher. Dietary intakes of vitamins B1, B2, B3, folate and B12 were not significantly associated with the incidence of advanced AMD (Table 2).

### 3.4. Multivariate Associations between Serum B Vitamins and Risk of AMD

Serum deficiency concerned 12.6% (<20 nmol/L), 7.6% (<10 nmol/L) and 10.2% (<185 pmol/L) for vitamins B6, folate and B12, respectively. Among participants who developed advanced AMD, 15.9% were deficient in folate versus 6.7% among participants who did not develop advanced AMD (Table 3).

**Figure 1:**
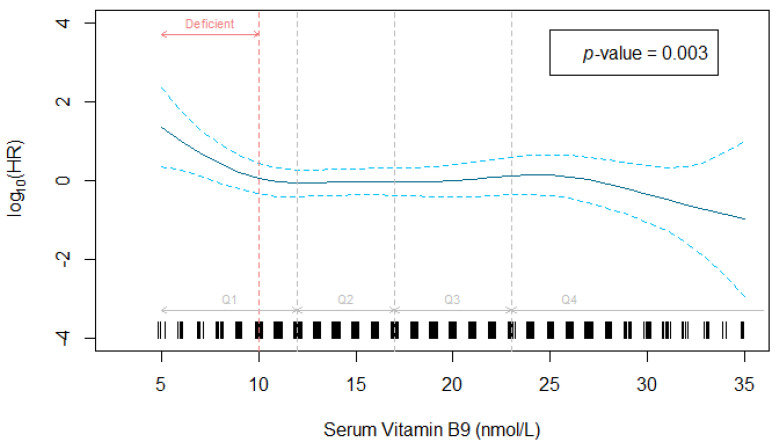
Association between serum folate and incidence of advanced AMD adjusted for age, sex, smoking status, HDL-cholesterol, genetic risk score, oral supplementation for AMD, body mass index and physical activity. Data from the Alienor Study 2001–2017 (*n* = 654). Serum folate was modeled using p-spline with 4 degrees of freedom in the Cox model. HR: hazard ratio.

Serum folate was significantly associated with a decreased risk of advanced AMD (*p* = 0.003, Figure 1). Since this association was not log-linear, the hazard function is represented using p-spline with 4 degrees of freedom, showing a decrease in the hazard of advanced AMD to about 10 nmol/L (corresponding to the threshold for deficiency), a plateau between 10 and 25 nmol/L and a further decrease above 25 nmol/L.

At any time after inclusion, participants with a normal status for folate (≥10 nmol/L) had a 2-fold lower hazard rate of advanced AMD compared with participants with a deficient status, after adjustment (Table 3).

In multivariable analyses, serum vitamins B6 and B12 were not significantly associated with the incidence of advanced AMD. The relationships between serum vitamins B6 and B12 and the risk of AMD did not depart significantly from log-linearity (Figure S5).

To assess the potential synergistic effect of these three vitamins, we estimated the association with AMD by adding vitamin B6, folate and B12 in the same adjusted-Cox model. After adjustment for serum B6 and B12 vitamins, serum folate was still significantly associated with AMD risk and the association for serum B6 and B12 remained unchanged.

## 4. Discussion

This study adds new understanding by documenting the prospective association between both dietary and serum B vitamins and the incidence of advanced AMD in a cohort of French older adults. To our knowledge, the present study is the first to demonstrate that a normal status for folate (≥10 nmol/L) was associated with lower rates of advanced AMD. This study also highlights that higher dietary intakes of vitamin B5 and B6 were associated with lower rates of advanced AMD.

Our results are in line with the Blue Mountains Eye Study (BMES), reporting that folate deficiency was significantly associated with higher 10-year incidence of early and any AMD [23], but not with advanced forms. Previous cross-sectional [42,43] and case–control [24,44] studies did not report significant associations for serum folate. Previous studies suggested the beneficial effect of high dietary intake of folate for reducing AMD risk [7,20,21,23]. In our study, dietary folate was not related to AMD.

The average dietary intake of folate in our cohort was 290 µg/d, which is lower than the average previously reported in the AREDS 1 and 2 studies (mean 383 µg/d in participants aged 55–85 years) [7] and the BMES study (440.8 µg/d for incident AMD and 432.5 µg/d for non-incident AMD, participants aged 55 or more) [23]. A major explanation could be food fortification. France does not have a folate-enriched food policy, thus in the present study, dietary intake is only provided by unfortified/natural food. Food folate has a lower bioavailability than folic acid added to foods or consumed as a supplement, which might contribute to this result and the lack of association between dietary folate and AMD in our study.

We reported a significant association with serum folate and no significant associations with folate evaluated from diet. As the diet was collected between 2 and 4 years after serum measurement, this might explain the observed differences between diet and serum results. Additionally, intake of folate was assessed with one 24 h recall, which is subject to participants’ memories; thus, serum measurement represents a more objective assessment of folate status. Our results regarding dietary vitamins B5 and B6 should also be interpreted with caution for the same reasons.

The biological mechanisms underlying the beneficial effect of folate on AMD risk are not well understood. One plausible explanation could be the potential role of B vitamins in DNA methylation processes. These nutrients play a key role in one-carbon metabolism and their deficiency could significantly reduce DNA methylation, leading to epigenome-dependent changes in the expression of disease-related factors [18,45,46]. Dysregulation in one-carbon metabolism are linked to many neurodegenerative and age-related diseases [46] and folate deficiency could have deleterious effects on cells by allowing homocysteine accumulation [45]. Hyperhomocysteinemia is a risk factor for many age-related diseases, and observational studies have shown that individuals with advanced AMD have higher serum homocysteine concentrations [13,23,24].

The association of serum vitamin B6 with AMD risk in our study was not significant. To our knowledge, no published study has reported results on serum vitamin B6. Our study suggested a decreased risk for AMD in participants with high B6 and B5 intakes but results from the literature are still unclear for B6 [7,19,20] and no study has reported results for the intake of B5 vitamin.

Association of serum vitamin B12 with AMD in our study was not significant. Results in the literature are conflicting. Some studies have previously reported significant associations between B12 deficiency and a higher rate of prevalent neovascular AMD [24], prevalent advanced forms [43] and 10-year incidence of advanced AMD [23], while results were not significant in other studies [42,44].

Regarding dietary intake of vitamin B1, B2, B3 and B12, our results are supported by previous results from AREDS 1 and 2 and BMES studies, which reported no associations between these vitamins and the risk of advanced AMD [7,20].

Residual confounding is a common limitation in epidemiologic studies, and the potential benefit of B vitamins might be explained by other factors. For instance, participants with high dietary and/or serum vitamin B are more likely to have a healthier lifestyle. In nutritional epidemiology, intercorrelations between nutrients cannot be completely eliminated. Folate is mainly provided by green vegetables, fruits, nuts, beans, and peas, and it shares common food sources with nutrients such as lutein, which has a protective effect on AMD risk [4,6,47,48]. We therefore adjusted for numerous diet- and AMD-related risk factors in order to minimize the possibility of residual confounding from unknown factors that may have influenced vitamin B exposures.

Assessment of dietary consumption is particularly difficult in humans. Dietary recall methods rely on the individuals’ memories and face the difficulties of the extreme day-to-day variability of the human diet, the bias in reporting because of social standards and nutritional recommendations, and the estimation of the nutritional content of foods. In particular, we collected a single 24 h recall. One 24 h recall cannot capture long-term dietary intake patterns for each individual because of high intra-individual variation and might lead to misclassification, especially for foods consumed occasionally (such as offal, a top source for vitamin B12). Misreporting (under- and over-reporting of energy intake) could also be a limitation of the 24 h recall. However, if sample sizes are sufficiently large, they may be used to determine the average intake in defined subgroups of a population [49].

Other limitations of our study include a single serum measurement that does not allow us to measure changes over time and could only reflect recent nutritional status.

Strengths of this study include its prospective design and long-term follow-up. This study used high quality retinal imaging reviewed by an independent committee of retina specialists to ensure negligible misclassification of incident AMD cases. Nutritional exposure was collected prior to the onset of AMD, and the potential for dietary changes resulting from knowledge of the disease or the induced disability was therefore minimized. Our study includes both dietary and serum measurements that are more objective and reproducible measurements of dietary status, limiting dietary assessment bias. Biomarkers also have the advantage to take into account the bioavailability.

## 5. Conclusions

In conclusion, this cohort study of French older adults suggests a strong association between a normal serum folate status, a high dietary intake of B5 and B6 and a lower risk for developing advanced AMD. Eating a healthy diet rich in vitamins B, particularly folate (leafy vegetables, fruits, whole grains), B5 (meat products, bread, milk-based products, vegetables) and B6 (liver, fish, leafy vegetables) may help to reduce vision loss due to advance forms of AMD.

## Figures and Tables

**Table 1:** Baseline population characteristics according to AMD status in the Alienor Study (*n* = 861, 2001–2017).

	Participants, No (%)			
Characteristics	Total (*n* = 861)	Incident AMD Cases (*n* = 93)	Non-Incident AMD Cases (*n* = 768)	*p*-Value ^a^
**Female**, sex	530 (61.6)	68 (73.1)	462 (60.2)	0.02
**Age**, mean (SD), y	74.7 (4.3)	76.4 (4.5)	74.5 (4.2)	<0.001
**Smoking status**, pack years				0.61
Never smoker	549 (64.4)	59 (64.1)	490 (64.5)	
<20	153 (18.0)	14 (15.2)	139 (18.3)	
≥20	150 (17.6)	19 (20.7)	131 (17.2)	
Missing data	9	1	8	
**Physical activity**				0.28
None	459 (53.3)	48 (51.6)	411 (53.5)	
Medium	173 (20.1)	18 (19.4)	155 (20.2)	
High	87 (10.1)	6 (6.5)	81 (10.5)	
No answer	142 (16.5)	21 (22.5)	121 (15.8)	
**Alcohol consumption**, mean (SD), g/day	12.0 (14.5)	12.0 (14.3)	12.0 (14.5)	0.98
Missing data	10	2	8	
**Use of AMD supplement**	98 (11.4)	27 (27.5)	71 (8.5)	<0.001
**Body mass index**, mean (SD), kg/m^2^	26.4 (3.9)	25.8 (3.6)	26.4 (4.0)	0.18
Missing data	5	0	5	
**HDL-Cholesterol**, mean (SD), mg/dL	61.9 (15.6)	65.8 (16.0)	61.5 (15.2)	0.01
Missing data	46	5	41	
**Genetic risk score**, mean (SD)	0.28 (1.19)	0.98 (1.35)	0.19 (1.14)	<0.001
Missing data	141	12	129	

Abbreviations: AMD, age-related macular degeneration; CI, confidence interval; HDL, high-density lipoprotein; HR, hazard ratio; SD, standard deviation. SI conversion factors: To convert HDL-cholesterol to mmol/L, multiply by 0.0259; **^a^** *p* value were obtained by using Chi^2^ analyses for categorical data and *t* test for continuous variables.

**Table 2:** Associations between dietary vitamins B and incidence of advanced AMD in the Alienor Study 2001–2017 ^a^.

Dietary Intake of Vitamins B	Participants, Total No.				
	Total (*n* = 710)	Incident AMD (*n* = 80)	Non-Incident AMD (*n* = 630)	HR ^b^ (95% CI)	*p* Value
Vitamin B1, mean (SD), mg/d	1.04 (0.44)	1.02 (0.43)	1.05 (0.45)	0.97 (0.74–1.27)	0.83
Vitamin B2, mean (SD), mg/d	1.60 (0.78)	1.52 (0.50)	1.61 (0.80)	0.78 (0.56–1.10)	0.15
Vitamin B3, mean (SD), mg/d	14.69 (7.01)	14.18 (6.34)	14.76 (7.09)	0.92 (0.70–1.21)	0.56
Vitamin B5, mean (SD), mg/d	4.20 (1.84)	3.88 (1.50)	4.24 (1.88)	0.72 (0.53–0.99)	0.049
Vitamin B6, mean (SD), mg/d	1.47 (0.60)	1.41 (0.57)	1.48 (0.60)	0.90 (0.81–0.99)	0.049
Folate, mean (SD), µg/d	290 (143)	302 (181)	288 (138)	1.02 (0.82–1.28)	0.83
Vitamin B12, mean (SD), µg/d	6.10 (12.78)	4.93 (8.05)	6.24 (13.25)	0.77 (0.51–1.17)	0.22

Abbreviations: AMD, age-related macular degeneration; CI, confidence interval; HR, hazard ratio; SD, standard deviation. ^a^ Cox proportional hazards regression model adjusted for age, sex, total energy intake, smoking status, HDL-cholesterol, genetic risk score, oral supplementation for AMD, body mass index and physical activity. ^b^ For 1-SD increase.

**Table 3:** Associations between serum vitamins B and incidence of advanced AMD in the Alienor Study 2001–2017.

Serum Level of Vitamins B	Total		Incident AMD		Non-Incident AMD		HR (95% CI)	*p* Value
	No.	Mean (SD) or (%)	No.	Mean (SD) or (%)	No.	Mean (SD) or (%)		
Vitamin B6, nmol/L	645	41.92 (36.41)	67	37.37 (27.73)	578	42.45 (37.27)	0.86 (0.59–1.25) ^a^	0.43
Deficient <20 nmol/L	81	(12.6)	13	(19.4)	68	(11.8)	Reference	
Normal ≥20 nmol/L	564	(87.4)	54	(81.6)	510	(88.2)	0.64 (0.5–1.20) ^a^	0.16
Folate, nmol/L	654	19.02 (9.97)	69	16.58 (7.09)	585	19.30 (10.22)	ND ^c^	
Deficient <10 nmol/L	50	(7.6)	11	(15.9)	39	(6.7)	Reference	
Normal ≥10 nmol/L	604	(92.4)	58	(84.1)	546	(93.3)	0.49 (0.25–0.95) ^b^	0.036
Vitamin B12, pmol/L	648	374 (431)	70	417 (545)	578	369 (416)	1.06 (0.89–1.27) ^a^	0.51
Deficient <185 pmol/L	66	(10.2)	5	(7.1)	61	(10.5)	Reference	
Normal ≥185 pmol/L	582	(89.8)	65	(92.9)	517	(89.5)	1.61 (0.64–4.06) ^a^	0.31

Abbreviations: AMD, age-related macular degeneration; CI, confidence interval; HR, hazard ratio; SD, standard deviation. ^a^ Cox proportional hazards regression model adjusted for age, sex, smoking status, HDL-cholesterol, genetic risk score, oral supplementation for AMD, body mass index, physical activity and alcohol consumption. HR variations are for 1-SD increase. ^b^ Cox proportional hazards regression model adjusted for age, sex, smoking status, HDL-cholesterol, genetic risk score, oral supplementation for AMD, body mass index and physical activity. ^c^ the association of serum folate with the risk of incident advanced was not log-linear and thus this HR cannot be estimated.

## Data Availability

The dataset presented in this article are not readily available because of ethical and legal re-strictions. Requests to access the dataset should be directed to the Steering Committee of the Alienor Study (contact cecile.delcourt@u-bordeaux.fr).

## Supplementary Materials

The following supporting information can be downloaded at: https://www.mdpi.com/article/10.3390/nu14142821/s1, Table S1. Comparison between included and excluded participants. Alienor Study (*n* = 963, 2001–2017); Figure S1. Alienor study timeline; Figure S2. Flow chart describing the sample selection from the Alienor Study 2001–2017; Figure S3: Description of dietary intake of B vitamins at inclusion among participants of the Alienor Study (2001, *n* = 861). Violin plots by gender; Figure S4: Dose–response relationships between dietary B vitamins and hazard ratios for advanced AMD estimated by penalized splines (4 ddl) in Cox proportional hazard models, Alienor Study, 2001–2017; Figure S5: Dose–response relationships between serum vitamins B6 and B12 and hazard ratios for advanced AMD estimated by penalized splines (4 ddl) in Cox proportional hazard models, Alienor Study, 2001–2017.
