# Supplementary material for: B Vitamins and Incidence of Advanced Age-Related Macular Degeneration: The Alienor Study
Source: Nutrients. 2022 Jul 8;14(14):2821. doi: 10.3390/nu14142821 (PMC9318446; doi:10.3390/nu14142821)
Supplement: Supplementary file 1 [file nutrients-14-02821-s001.zip › nutrients-1789415-supplementary.pdf]

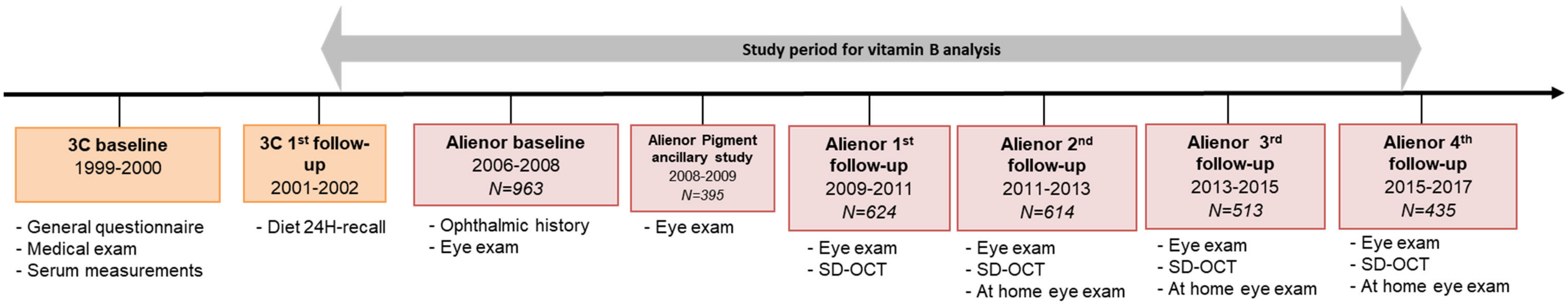

Figure S1. Alienor study timeline.

AMD: age-related macular degeneration; SD-OCT: spectral domain optical coherence tomography.

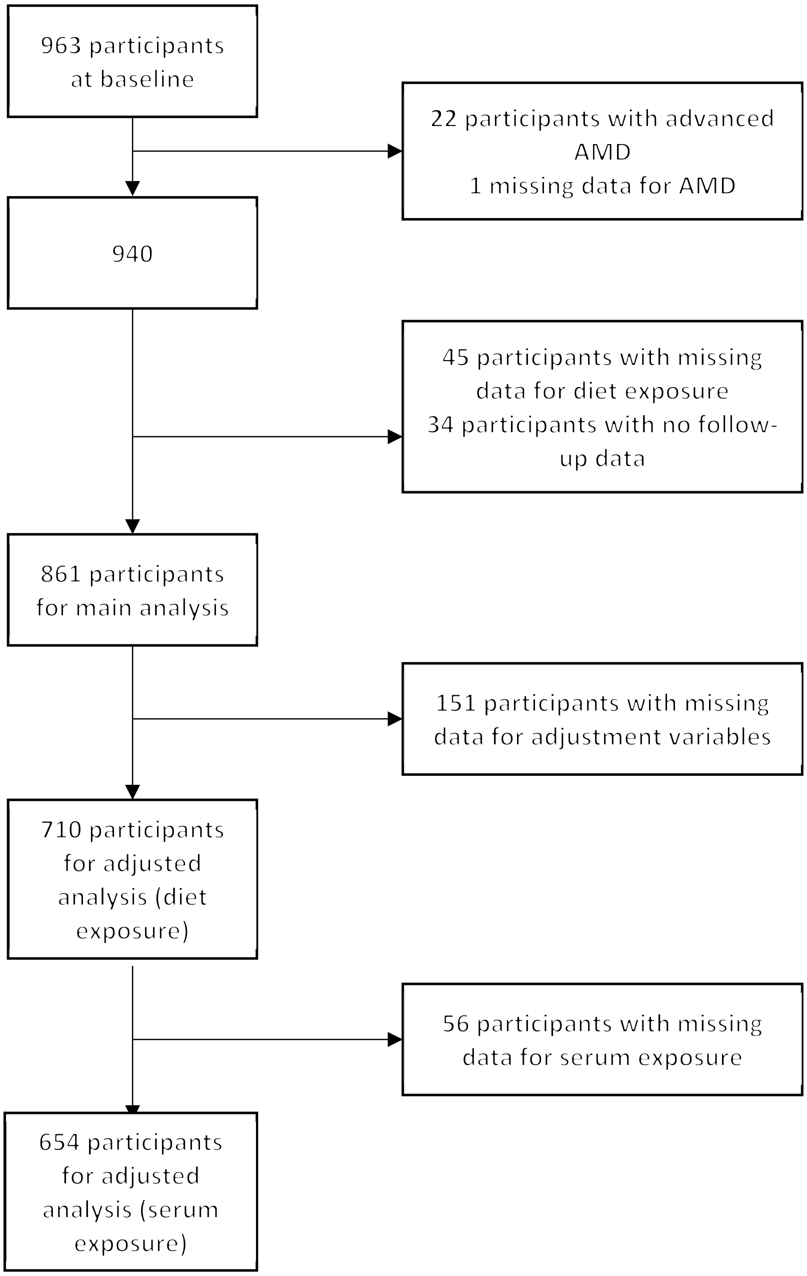

Figure S2. Flow chart describing the sample selection from the Alienor Study 2001-2017.

AMD: age-related macular degeneration;

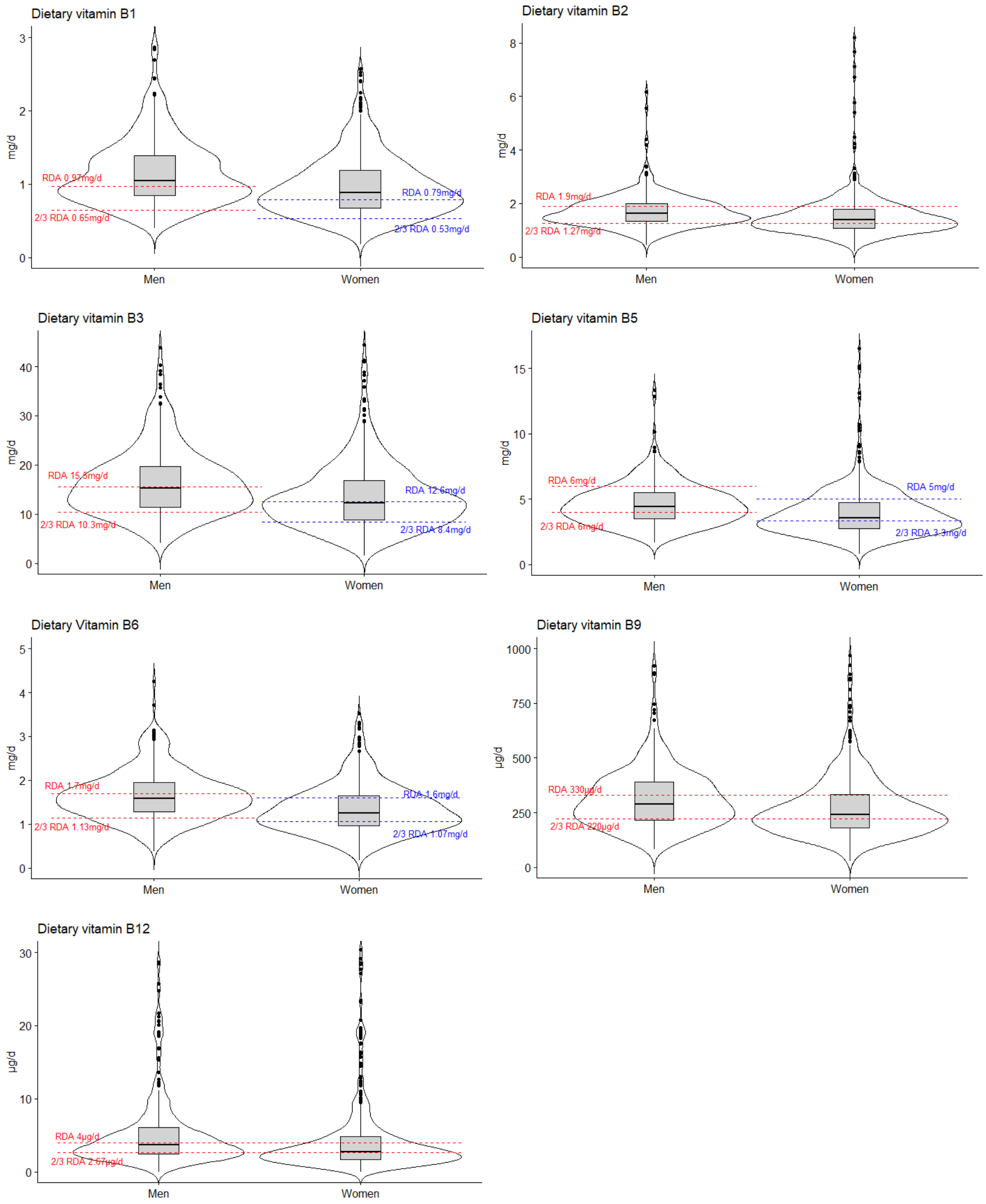

Figure S3: Description of dietary intake of B vitamins at inclusion among participants of the Alienor Study (2001, N=861). Violin plots by gender.

RDA: Recommended dietary allowance for the French population.

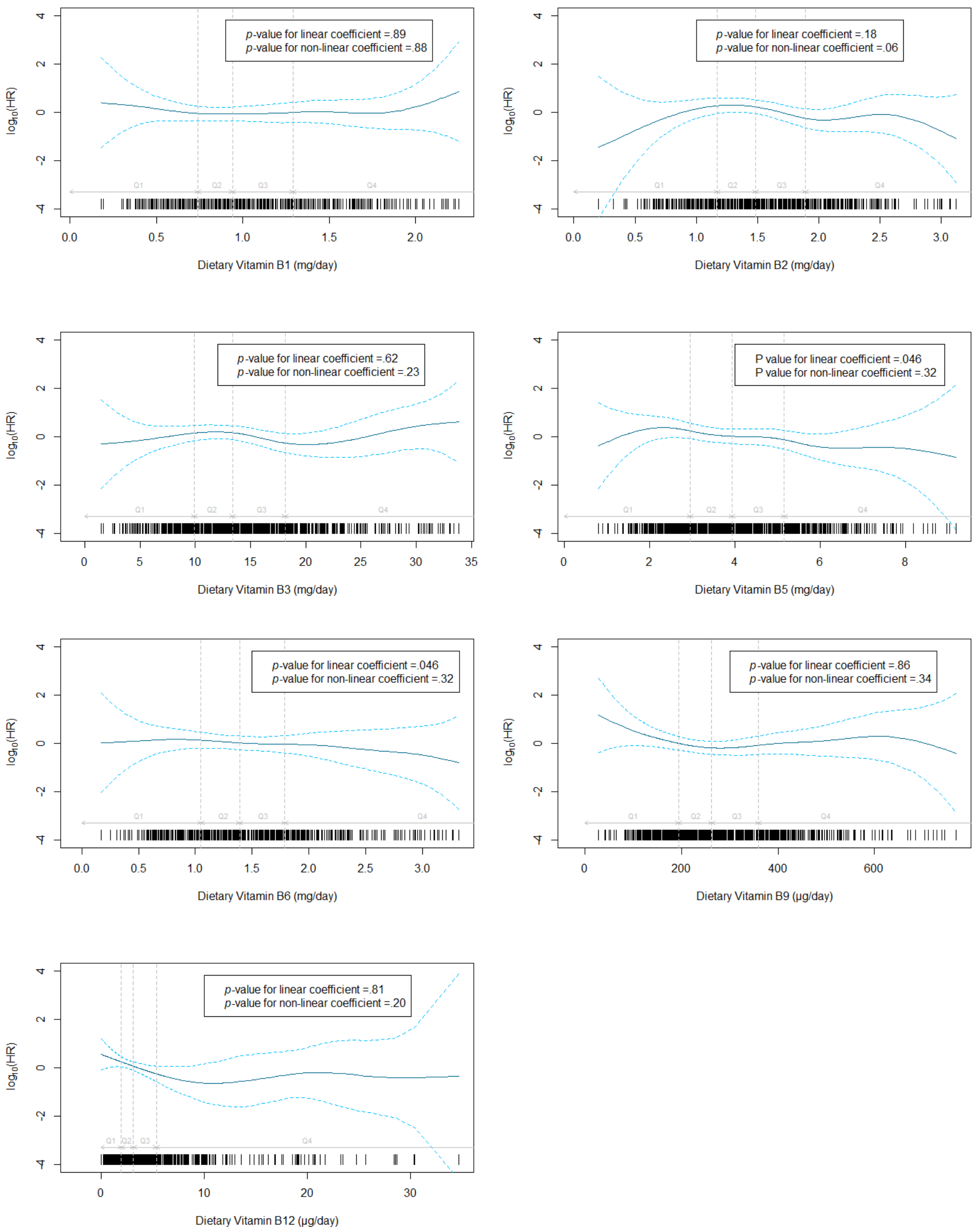

Figure S4: Dose-response relationships between dietary B vitamins and hazard ratios for advanced AMD estimated by penalized splines (4 ddl) in Cox proportional hazard models, Alienor Study, 2001-2017.

p-value for linear coefficient is the *p*-value for the test of a linear association against a null association. *p*-value for non-linear coefficient is the *p*-value for the test of a non-linear association against a linear association. Vertical dashed lines indicate quintiles of distribution for B vitamins.

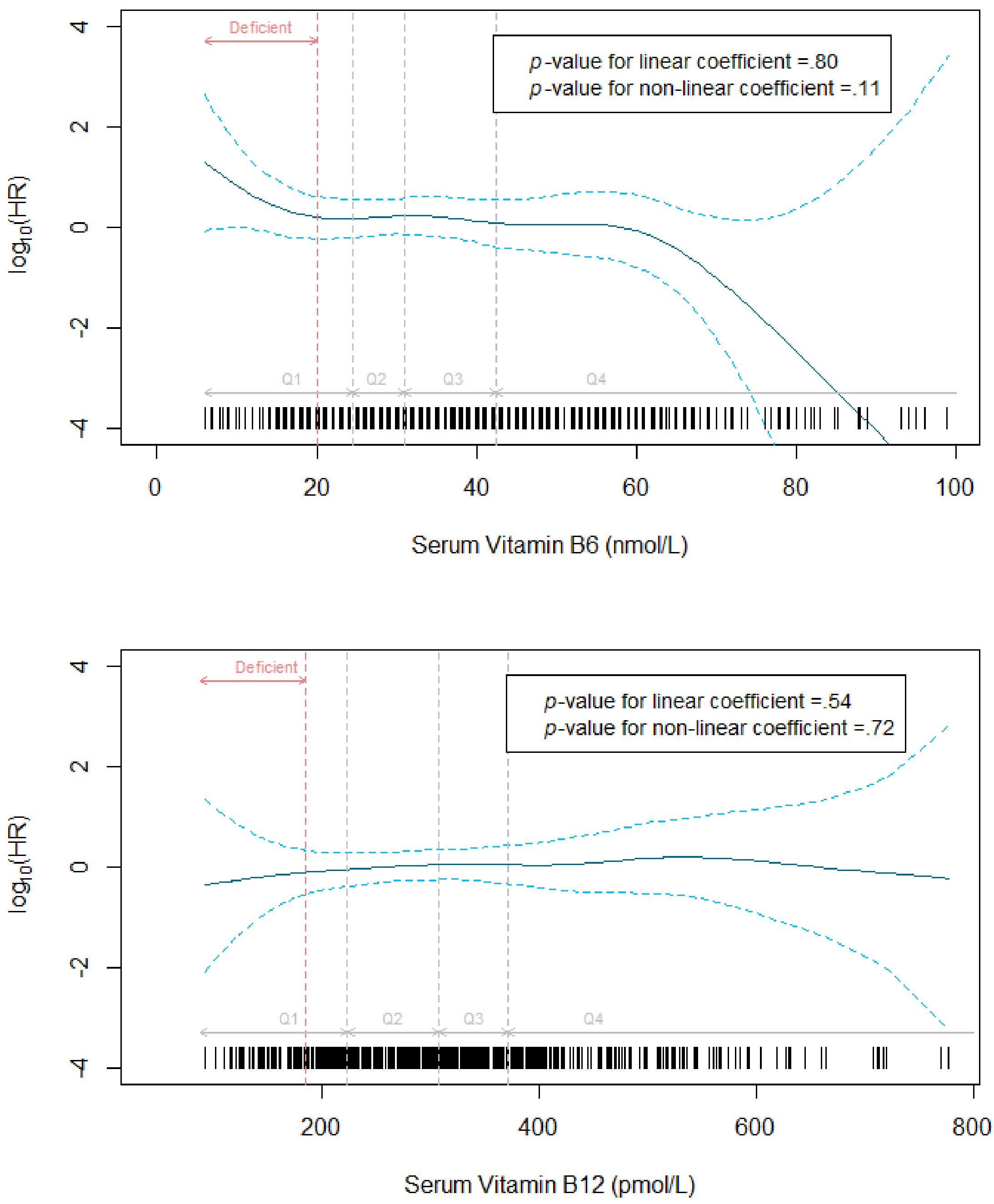

Figure S5: Dose-response relationships between serum vitamins B6 and B12 and hazard ratios for advanced AMD estimated by penalized splines (4 ddl) in Cox proportional hazard models, Alienor Study, 2001-2017.

p-value for linear coefficient is the *p*-value for the test of a linear association against a null association. *p*-value for non-linear coefficient is the *p*-value for the test of a non-linear association against a linear association. Vertical dashed lines indicate quintiles of distribution for B vitamins.

Table S1. Comparison between included and excluded participants. Alienor Study (N=963, 2001-2017).

	Participants, No (%)		
**Characteristics**	**Included (n=861)**	**Excluded (n=102)**	**P-Value[a]**
Female, sex	530 (61.6)	66 (64.7)	0.54
Age, mean (SD), y	74.7 (4.3)	78.9 (3.8)	<0.001
Missing data	0	50	
Smoking status, pack-years			0.64
Never smoker	549 (64.4)	65 (65.7)	
<20	153 (18.0)	20 (20.2)	
≥20	150 (17.6)	14 (14.1)	
Missing data	9	3	
Physical activity			<0.001
None	459 (53.3)	46 (45.1)	
Medium	173 (20.1)	17 (16.7)	
High	87 (10.1)	3 (2.9)	
No answer	142 (16.5)	36 (35.3)	
Alcohol consumption, mean (SD), g/day	12.0 (14.5)	10.3 (12.0)	0.23
Missing data	10	12	
Use of AMD supplement	98 (11.4)	13 (12.7)	0.68
Body mass index, mean (SD), kg/m²	26.4 (3.9)	26.3 (3.8)	0.84
Missing data	5	3	
HDL-Cholesterol, mean (SD), mgl/dL	61.9 (15.6)	64.7 (16.0)	0.66
Missing data	46	16	
Genetic risk score, mean (SD)	0.28 (1.19)	0.67 (1.25)	0.01
Missing data	141	25	

Abbreviations: AMD, age-related macular degeneration; HDL, high-density lipoprotein

SI conversion factors: To convert HDL-cholesterol to mmol/L, multiply by 0.0259

[a] p value were obtained by using Chi² analyses for categorical data and *t* test for continuous variables.
